# Binding of Carbonic Anhydrase IX to 45S rDNA Genes Is Prevented by Exportin-1 in Hypoxic Cells

**DOI:** 10.1155/2015/674920

**Published:** 2015-02-22

**Authors:** Emanuele Sasso, Monica Vitale, Francesca Monteleone, Francesca Ludovica Boffo, Margherita Santoriello, Daniela Sarnataro, Corrado Garbi, Mariangela Sabatella, Bianca Crifò, Luca Alfredo Paolella, Giuseppina Minopoli, Jean-Yves Winum, Nicola Zambrano

**Affiliations:** ^1^Dipartimento di Medicina Molecolare e Biotecnologie Mediche, Università di Napoli Federico II, 80131 Napoli, Italy; ^2^CEINGE Biotecnologie Avanzate, Via Gaetano Salvatore 486, 80145 Napoli, Italy; ^3^Associazione Culturale DiSciMuS RFC, 80026 Casoria, Italy; ^4^Institut des Biomolécules Max Mousseron, UMR 5247 CNRS, Université Montpellier I & II, ENSCM, 34296 Montpellier, France

## Abstract

Carbonic anhydrase IX (CA IX) is a surrogate marker of hypoxia, involved in survival and pH regulation in hypoxic cells. We have recently characterized its interactome, describing a set of proteins interacting with CA IX, mainly in hypoxic cells, including several members of the nucleocytoplasmic shuttling apparatuses. Accordingly, we described complex subcellular localization for this enzyme in human cells, as well as the redistribution of a carbonic anhydrase IX pool to nucleoli during hypoxia. Starting from this evidence, we analyzed the possible contribution of carbonic anhydrase IX to transcription of the 45S rDNA genes, a process occurring in nucleoli. We highlighted the binding of carbonic anhydrase IX to nucleolar chromatin, which is regulated by oxygen levels. In fact, CA IX was found on 45S rDNA gene promoters in normoxic cells and less represented on these sites, in hypoxic cells and in cells subjected to acetazolamide-induced acidosis. Both conditions were associated with increased representation of carbonic anhydrase IX/exportin-1 complexes in nucleoli. 45S rRNA transcript levels were accordingly downrepresented. Inhibition of nuclear export by leptomycin B suggests a model in which exportin-1 acts as a decoy, in hypoxic cells, preventing carbonic anhydrase IX association with 45S rDNA gene promoters.

## 1. Introduction

Reprogrammed energy metabolism was considered among the emerging hallmarks in cancer [1]. Cancer cells developing inside a hypoxic environment, but also cancer cells exposed to normal oxygen levels, switch energetic metabolism towards glycolysis. Thus, gene expression programmes mediated by HIF1*α* transcription factor allow cells to increase the efficiency of glycolysis via enhanced ability to uptake glucose, via stimulation of glycolytic enzymes, and via increased ability to buffer the acidic, pyruvate- and lactate-enriched intracellular environment. The carbonic anhydrases (CAs), a large family of metalloenzymes with wide subcellular distributions, are central to the adaptation of the cancer cells to the glycolytic switch. Namely, CA IX, a membrane carbonic anhydrase possessing an extracellular catalytic domain, is actively involved in the acidification of extracellular space, as a consequence of the need for buffering the intracellular compartments [2, 3]. Cancer cells may also take advantage from the acidic features of their extracellular space, since it may enhance invasiveness potential [4].

CA IX structure was recently defined [5]; these authors proposed a dimeric assembly for the enzyme, exposing a highly glycosylated proteoglycan-like domain and the catalytic domain towards the extracellular compartment, and a short C-terminal tail exposed to the intracellular environment. These regions are separated by a single transmembrane helical region. An intact intracellular domain is required for extracellular acidification by CA IX [6], implying that its interactions with intracellular proteins are fundamental for biological properties. Due to its ability to participate in the adaptation of the cancer cells to the metabolic stresses, CA IX is actively involved in cancer cell survival [7]. This renders CA IX a cancer biomarker for prognosis and resistance to treatments [8] and an attractive target of therapy. Several classes of inhibitors are currently available to target CAs: among these, sulfonamides and derivatives, acting as metal ion binders; compounds, such as phenols, polyamines, esters, carboxylates, and sulfocoumarins, possessing the ability to anchor to the zinc-coordinated water molecule/hydroxide ion; coumarin and related compounds which bind at the entrance of CA active site [9, 10]. Current efforts in the design and exploitation of selective CA inhibitors deal with the structure-based rational search [11–13] and with their potential as agents sensitizing to combined treatments in cancer [14].

The characterization of protein interactomes is a potent tool to discover and annotate protein functions in cellular physiology and in disease [15], as well as for the design of tumour-targeting peptides and mimetics [16]. We have recently annotated the CA IX interactome [17], highlighting the hypoxia-regulated interaction of CA IX with a list of components of the nuclear import and export machineries. These proteins also shared HEAT/ARM repeat protein domains. Additional intracellular proteins were also able to bind CA IX, such as CAND1, in an interaction occurring also in normoxic cells. The C-terminal region of CA IX was also shown to be necessary and sufficient for these interactions. In agreement with these results, immunofluorescence analysis in permeabilized cells showed a complex subcellular distribution for CA IX, which appeared to be widely distributed in normoxic and hypoxic mammalian cells of different origin. Interestingly, a pool of CA IX and of one of its main interactors, exportin-1 (XPO1), was clearly redistributed to perinuclear regions and nucleoli as a consequence of hypoxia. Finally, occurrence of CA IX in nuclear and/or perinuclear compartments was also highlighted in cases of clear-cell kidney carcinomas [7, 17], confirming previous evidences, describing nuclear CA IX in tumours characterized by poor prognosis [18, 19]. Taken together, these evidences can extend the classical view of CA IX as a cell surface protein, towards a concept of intracellular signalling component and multifunctional effector in cellular physiology and cancer biology. Accordingly, experimental evidences support a constitutive shedding of CA IX ectodomain, which may regulate surface availability of the protein, but also signalling properties of the released N- and C-terminal protein domains both in the extracellular and in the intracellular compartments, respectively [20]. CA IX is also actively internalized in the endocytic pathways, the latter being a route for CA IX redistribution in intracellular compartments, including perinuclear regions and nuclei.

Nucleolus is the organelle for ribosomal 45S rRNA precursor synthesis and processing, as well as for ribosome biogenesis (see [21] for a recent review). 45S rDNA genes are present in 300–400 copies in mammalian genomes. They are typically arranged in tandemly repeated arrays at few chromosome* loci*. 45S rDNA genes can also show different chromatin states, which well correlate with the expression state, thus justifying the occurrence of epigenetic mechanisms underlying transcriptional activity. However, nucleoli are also the sites for regulated sequestration and release of important signalling proteins, such as those modulating p53 activity, such as MDM2 [22]. Thus, nucleoli are currently believed to be multifunctional organelles and modulators of cellular responses to stresses. Cells also cope with nucleolar stress, so that overcoming nucleolar stress can be viewed as an emerging hallmark in cancer [23].

In this paper, we report on a novel, putative nucleolar function for CA IX, since we demonstrate binding of the protein to the 45S rDNA genes in normoxic cells. We also show that hypoxic stimulation releases CA IX from occupancy of the nucleolar chromatin, releasing the protein, which becomes part of complexes with XPO1. Concurrently, 45S rRNA transcript levels are decreased, supporting a functional role, for CA IX, as a regulator of transcription for rDNA genes.

## 2. Materials and Methods

### 2.1. Cell Cultures and Manipulations, DNA Constructs

The HEK293 and SHSY-5Y cell lines were purchased from ATCC. Cells were cultured in standard conditions using DMEM complemented with 1% penicillin/streptomycin, 2 mM glutamine, and 10% fetal bovine serum (Euroclone), at 37°C, in 5% CO_2_ humidified atmosphere.

Putative and canonical NLS and NES sequences were frame-fused at the C-terminus of EGFP in the vector of expression pEGFP_C1. The corresponding primers were the oligonucleotides 1 and 2 for the construct pEGFP_NLS-SV40 (TAg) and the oligonucleotides 3 and 4 for pEGFP_NES-PKIA. The construct pEGFP_CA IX putative NLS, encompassing sequence from amino acids 434 to 459 of the full length protein, was generated by PCR from cDNA of full length CA IX using the oligonucleotides 5 and 6. The construct pEGFP_CA IX putative NES, including CA IX sequence from amino acid positions 412 to 429, was produced by annealing of the synthetic oligonucleotides 7 and 8.

 Oligo 1, NLS_SV40(TAg)_US: 5′-GATCTCCAAAAAAGAAGAGAAAGGTAG-3′;
 Oligo 2, NLS_SV40 (TAg)_LS: 5′-TCGACTACCTTTCTCTTCTTTTTTGGA-3′;
 Oligo 3, NES_PKIA_US: 5′-GATCTTTAGCCTTGAAATTAGCAGGTCTTGATATCG-3′;
 Oligo 4, NES_PKIA_LS: 5′-TCGACGATATCAAGACCTGCTAATTTCAAGGCTAAA -3′;
 Oligo 5, CA9_Cterm_For: 5′-ATAAGATCTCAGATGAGAAGGCAGCACAGA-3′;
 Oligo 6, CA9_Cterm_Rev: 5′-ACTGTAGTCGACGGCTCCAGTCTCGGCTACCT-3′;
 Oligo 7, CA9_NES_FWD: 5′-GATCTGCTGGTGACATCCTAGCCCTGGTTTTTGGCCTCCTTTTTGCTGTCACCAGCG-3′;
 Oligo 8, CA9_NES_RV: 5′-TCGACGCTGGTGACAGCAAAAAGGAGGCCAAAAACCAGGGCTAGGATGTCACCAGCA-3^′.^

All the primers were synthesized at CEINGE Biotecnologie Avanzate. The analysis of putative and canonical NES and NLS sequences was performed in HEK293 and SHSY-5Y cells after transfection of the previously described constructs with the calcium phosphate method [24]. Leptomycin B (Sigma Aldrich) was dissolved in 70% methanol (v/v); treatments with solvent or leptomycin B (20 ng/mL) were performed for 4 hours. Hypoxic treatments were performed for six hours, in an incubator (STEMCELL Technologies), with atmosphere containing 95% N_2_ and 5% CO_2_. CA inhibitor acetazolamide was dissolved in DMSO and challenged to cell cultures for 16 hours at 1 mM concentration. Vehicle DMSO was used as a carrier control at 0.1% v/v.

### 2.2. Antibodies, Fluorescence Microscopy, and Immunological Methods

The antibodies used in this study were M75 and VII-20 anti-CA IX mouse monoclonals [25]; anti-XPO1 goat polyclonal (CRM1 C-20, Santa Cruz Biotechnology); anti-UBF mouse monoclonal (F-9, Santa Cruz Biotechnology); anti-HIF1*α* mouse monoclonal (BD Transduction Laboratories); and anti-*β*-actin mouse monoclonal (AC-15, Santa Cruz Biotechnology). Western blot analysis was carried out on cellular lysates obtained as described [26]; the latter were resolved on 10% SDS-PAGE gels and transferred to PVDF membranes (Millipore). Filters were probed with the anti-HIF1*α* antibody or with the M75 antibody for CA IX and the AC-15 antibody for *β*-actin, followed by anti-mouse secondary antibody.

For fluorescence analysis of the EGFP fusion constructs, cells were fixed with 3% (w/v) paraformaldehyde and 1% (w/v) sucrose in PBS for 20 minutes at room temperature (RT). Immunofluorescence analysis was carried out, as described [17], with VII-20 anti-CA IX mouse monoclonal [25] and with anti-XPO1 goat polyclonal (CRM1 C-20, Santa Cruz Biotechnology). Samples were observed on a Zeiss LM510 confocal microscope.

About 1 × 10^7^ cells from triplicate cultures were used for each chromatin immunoprecipitation assay, which were fixed with 1% formaldehyde for 10 minutes at RT. Glycine 125 mM was added to inactivate the excess of formaldehyde. Chromatin was sonicated in a way to enrich the DNA fragments in the 200–1,000 bp size. Soluble fraction of chromatin was extracted by centrifugation and it was immunoprecipitated using CA IX VII-20, UBF1 (F-9, Santa Cruz) antibodies, and mouse IgGs as control.

### 2.3. Quantitative PCR Analysis

Triplicate sets of samples were used for quantitative PCR analyses (ChIP and qRT-PCR). Statistical analyses were carried out according to Student's *t*-test. Real-time PCR was used to detect enrichments of immunoprecipitated DNA in relation to total input chromatin. Supernatant obtained without antibody was used as input control. All the primers were used to a final concentration of 0,5 *μ*M in a 20 *μ*L real-time reaction containing 10 *μ*L of SYBR Green (Applied Biosystem) and 2 *μ*L DNA. The results were expressed as percent of total chromatin according to the following formula: 2ΔCt × 10, where Ct represents the cycle threshold and ΔCt = Ct (input) − Ct (immunoprecipitation).

Oligonucleotide sequences used to amplify the 45S rDNA region of interest were the following: 5′-GGTATATCTTTCGCTCCGAG-3′ and 5′-AGCGACAGGTCGCCAGAGGA-3′. The region of amplification was located between the promoter region and the start site of rDNA [27]. To analyze the relative abundance of cellular RNAs, quantitative RT-PCR was performed as described [28] on individual biological triplicates for each sample. Cells were lysed by Trizol (Euroclone), and the total RNA was extracted with phenol/chloroform. DNase-treated total RNA was reverse-transcribed (Im-Prom II, Promega), and then it was amplified in a 7500 Real-Time PCR System (Applied Biosystem) using SYBR Green PCR MASTERMIX (Applied Biosystem). All primers were used to a final concentration of 0,2 *μ*M. The oligonucleotide primers were designed using the bioinformatic tool Primer-BLAST (NCBI/ Primer-BLAST). Their sequences were the following: For_CAIX: 5′-CGGAAGAAAACAGTGCCTATGA-3′; Rev_CAIX: 5′-CTTCCTCAGCGATTTCTTCCA-3′; For_rRNA45S: 5′-CTCCGTTATGGTAGCGCTGC-3′; Rev_rRNA45S; 5′-GCGGAACCCTCGCTTCTC-3′; hLDHA_FOR: 5′-TGGCCTGTGCCATCAGTATC-3′; hLDHA_REV: 5′-CGATGACATCAACAAGAGCAAGT-3′; BActin_FOR: 5′-CGTGCTGCTGACCGAGG-3′; BActin_REV: 5′-GAAGGTCTCAAACATGATCTGGGT-3′. The relative abundance of each RNA was evaluated in relation to ACTB (*β*-actin) transcripts by ΔΔCt method [29, 30].

## 3. Results and Discussion

### 3.1. Putative NLS and NES Sequences in CA IX Direct the Nuclear Trafficking of EGFP in Human Cell Lines

**Figure 1:**
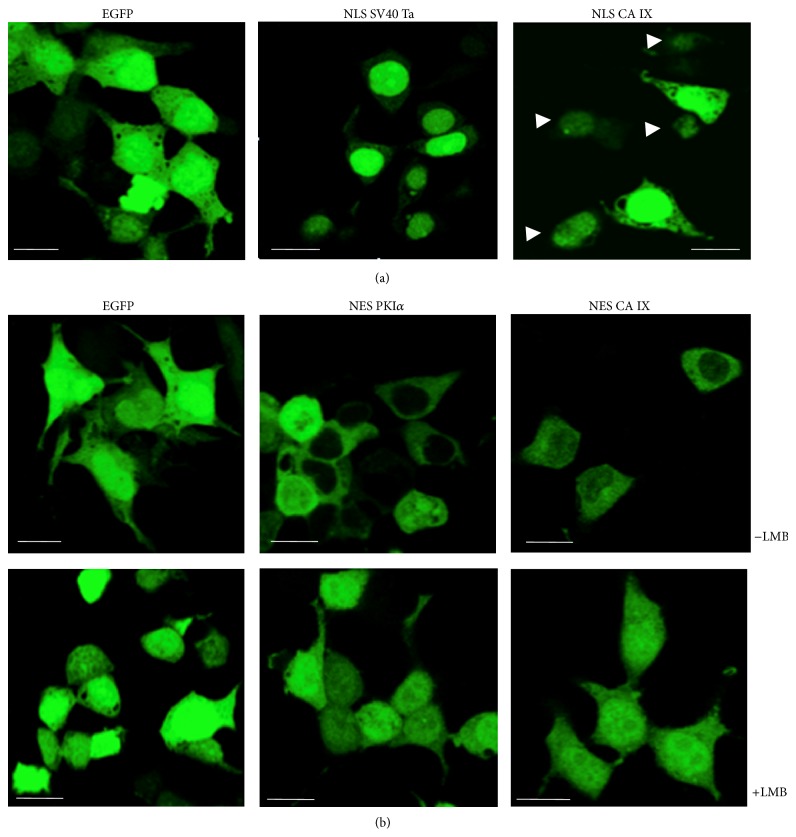
Analysis of the putative nuclear localization and nuclear export signals in CA IX. (a) EGFP protein or its fusions with a canonical NLS from SV40 Ta or with the region encompassing the putative NLS of the CA IX protein sequence (amino acid positions 434–459), as indicated, were expressed in HEK293 cells and visualized by confocal fluorescence microscopy. Arrowheads indicate cells, in which the EGFP protein fused to the putative CA IX NLS was almost exclusively nuclear. White bars: 10 *μ*m. (b) In the upper panels, EGFP protein or its fusions with a canonical NES from PKI*α* or with the putative NES-containing region of the CA IX protein sequence (amino acid positions 412–429), as indicated, were expressed in HEK293 cells and visualized by confocal fluorescence microscopy. In the lower panels, transfected cells were treated with leptomycin B (LMB) to inhibit XPO1-mediated nuclear export. White bars: 10 *μ*m.

In a previous study we described the interaction of CA IX with the cellular machinery of nuclear import and export [17]. CA IX was indeed found in native complexes with the importin, TNPO1, and the exportin, XPO1; accordingly, CA IX appeared to be widely distributed in the cellular compartments, including nuclei, of several human cell lines. Putative NLS and NES sequences in the CA IX sequence interacting with these proteins were predicted by bioinformatic tools. Thus, we evaluated the function of the putative NES and NLS signals, exploring their ability to direct the subcellular localization of EGFP, used as a reporter. In particular, the function of the putative NLS and NES sequences in CA IX was analyzed comparing the subcellular distribution in HEK293 cells of the isolated EGFP protein and of EGFP fusions bearing either canonical NES and NLS sequences or the putative NES and NLS sequences of CA IX.  Figure 1(a) shows the subcellular distributions of isolated EGFP (left panel), of EGFP fused to the reference NLS of the SV40 T antigen (middle) and of EGFP fused to the putative NLS of CA IX (right panel). While the isolated EGFP was distributed widely in the transfected cells, the putative NLS signal of CA IX led to some nuclear accumulation of the protein, despite a lower extent, compared to the NLS of SV40 Ta. In a complementary manner, as shown in the upper panels of Figure 1(b), the reference PKI*α* (middle) and the region containing the CA IX putative NES (right panel) sequences led to evident nuclear exclusion of the EGFP fluorescent proteins, compared to the isolated EGFP (left).

In order to confirm the actual function of the putative CA IX NES signal, the cells transfected with the various constructs were treated with leptomycin B, an inhibitor of nuclear export mediated by XPO1 [31]. Images (lower panels of Figure 1(b)) reveal that the isolated EGFP did not change its distribution (left) in the presence of leptomycin, while a clear redistribution in nuclear compartments was appreciated for the fluorescent PKI*α* NES (middle) and for the region containing the CA IX putative NES (right panel), in comparison to the untreated cells (upper panels). Similar results were obtained in neuroblastoma SHSY-5Y cells (data not shown). Thus, the putative NLS and, to a major extent, the NES sequence in CA IX are actually functional. This indeed suggests that the nuclear trafficking of CA IX and its putative function are dependent on the binding to its interactors. Taken together, these results support a nuclear function for CA IX in human cells.

### 3.2. CA IX Is Bound to Nucleolar Chromatin in Human Cell Lines

**Figure 2:**
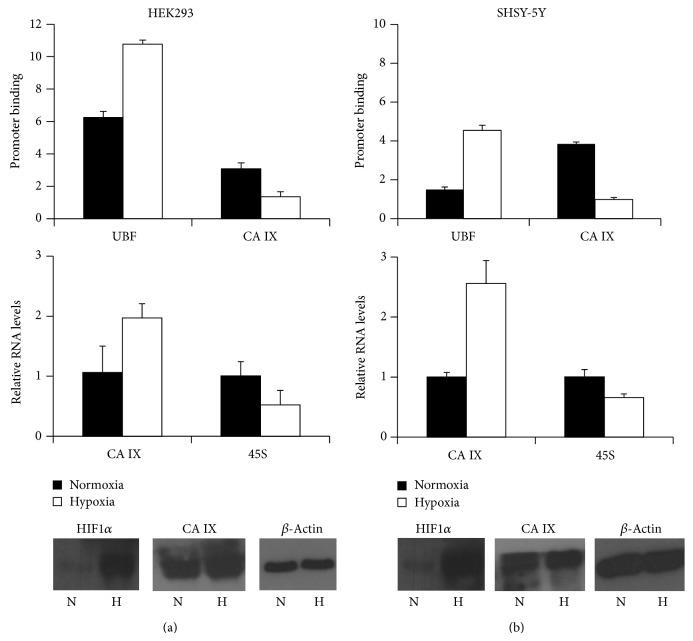
Chromatin immunoprecipitation analysis of CA IX and UBF1 binding to 45S rDNA precursor gene clusters. Normoxic or hypoxic HEK293 (a) and SHSY-5Y (b) cells were subjected to ChIP analysis with UBF1 or CA IX monoclonal antibodies, as indicated (upper charts). Binding of the proteins to 45S rDNA genes was evaluated via quantitative PCR analysis from triplicate samples. Calculated *P* values for pairwise comparisons of ChIP data were in the 0.0038 to 0.03 range (Student's *t*-test). The middle panels show the results of qRT-PCR analysis of CA IX or 45S rRNA transcripts from normoxic or hypoxic HEK293 (a) and SHSY-5Y (b) cells. Calculated *P* values for pairwise comparisons of qRT-PCR data were in the 0.0027 to 0.04 range. Lower panels show the hypoxia-induced increases in HIF1*α* and CA IX protein levels in normoxic (N) or hypoxic (H) HEK293 (a) and SHSY-5Y (b) cells. Filters were reprobed to *β*-actin, which was used as a loading control.

One additional feature, emerging from our previous analysis of CA IX interactome, was the increased abundance of CA IX/XPO1 complexes and their peculiar enrichment in nucleoli in hypoxic cells. Thus, we hypothesized a nucleolar function for CA IX and for its complexes with XPO1. Nucleoli are the districts in which 45S rRNA synthesis and processing occur to allow ribosome biogenesis. 45S rRNA precursor is actively transcribed from arrays of 45S rDNA genes in nucleoli. We then evaluated, by chromatin immunoprecipitation (ChIP) analysis, the potential binding of CA IX to nucleolar chromatin in normoxic and hypoxic HEK293 and SHSY-5Y cells. ChIP analysis was also performed for UBF1, an architectural factor regulating RNA polymerase I transcription. The data shown in the panel A of Figure 2 demonstrated that both UBF1 and CA IX are actually bound to rDNA 45S genes in normoxic HEK293 cells. Even in the presence of increases in CA IX protein levels (lower panels of Figure 2(a)), the exposure of the cells to a hypoxic environment resulted in a decreased association of CA IX to nucleolar chromatin, while UBF1 was stabilized on 45S rDNA genes. This was associated with decreased levels of 45S rRNA in hypoxic cells, as shown by the results of qRT-PCR in the middle panel of Figure 2(a). These data were consistently replicated in SHSY-5Y cells (Figure 2(b)). As previously demonstrated [17], CA IX levels and its nucleolar presence are increased in hypoxic cells, as for its participation in molecular complexes with XPO1. These novel results show that the increased formation of CA IX/XPO1 complex in the nucleoli of hypoxic cells is indeed associated with a decreased representation of CA IX on nucleolar chromatin and with a decreased transcription of 45S rDNA genes, both in HEK293 and in SHSY-5Y cells. These data support the hypothesis that in human cell lines CA IX might be associated with active transcription of 45S rDNA genes in normoxic condition.

### 3.3. Binding of CA IX to Nucleolar Chromatin Is Regulated by Its Interaction with XPO1 and by CA Activity

**Figure 3:**
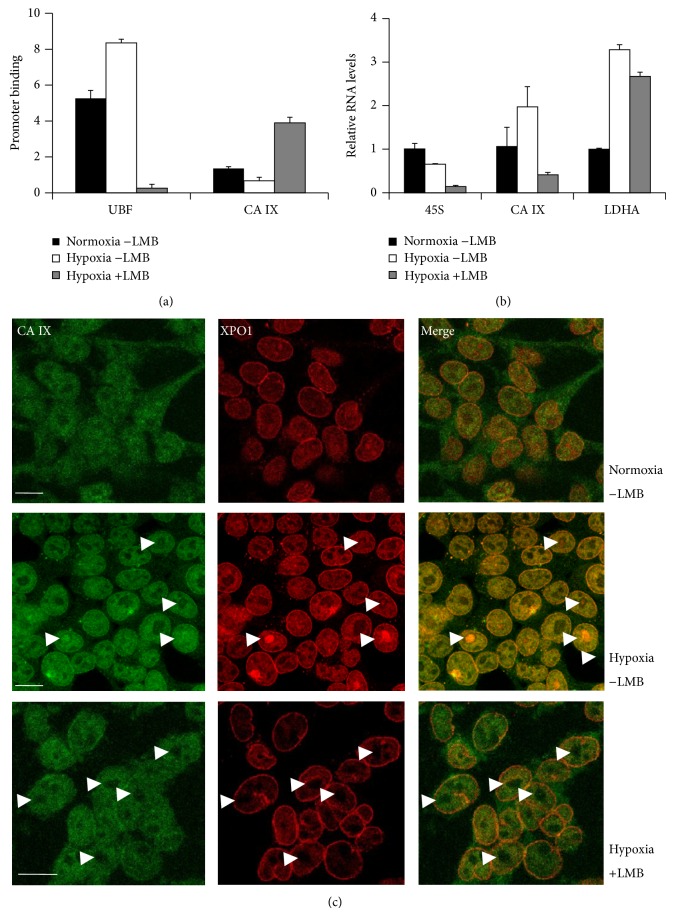
XPO1 controls binding of CA IX to 45S rDNA gene promoters. (a) Chromatin immunoprecipitation analysis of CA IX and UBF1 binding to 45S rDNA precursor gene clusters in a triplicate set of samples of normoxic and hypoxic HEK293 cells. Hypoxic cells were also exposed to leptomycin B (LMB) for 4 hours. Calculated *P* values for pairwise comparisons of ChIP data were in the 0.001 to 0.01 range (Student's *t*-test). (b) HEK293 cells from a triplicate set of samples exposed to normoxia, hypoxia, and hypoxia in the presence of leptomycin B were lysed for RNA extraction and qRT-PCR analysis of CA IX and LDHA mRNAs and 45S rRNA transcripts, as indicated. Calculated *P* values for pairwise comparisons of qRT-PCR data were in the 0.0005 to 0.01 range, with the exception of the CA IX transcript levels in normoxic and hypoxic/+LMB condition (*P* = 0.4, not significant) (Student's *t*-test). (c) Cells subjected to the same treatments, as in (a) and (b), were fixed, permeabilized, and exposed to antibodies for CA IX (green) and XPO1 (red) immunofluorescence analysis. Confocal images from representative fields were taken. White arrowheads in hypoxic cells (middle panels) indicate strong colocalization of CA IX and XPO1 in some nucleolar and subnucleolar areas. White arrowheads in hypoxic cells treated with leptomycin B (LMB) indicate the nucleoli, devoid of CA IX/XPO1 complexes. White bars: 10 *μ*m.

The data obtained so far support the hypothesis that CA IX acts as a positive modulator of transcription for rDNA 45S genes. In fact, decreased CA IX binding to nucleolar chromatin observed in hypoxic cells was associated with decreased 45S rRNA precursor transcript levels. What was also occurring in hypoxic cells was the increased representation of CA IX/XPO1 complexes in nucleoli [17]. Thus, XPO1 and its interaction with CA IX in the nucleoli of hypoxic cells may act as a decoy mechanism to prevent 45S rDNA genes' transcription during hypoxia. To validate this hypothesis, we evaluated 45S rDNA promoter binding by CA IX, 45S rRNA transcript levels, and CA IX/XPO1 colocalization in hypoxic HEK293 cells treated with the XPO1 inhibitor leptomycin B. As shown in Figure 3, CA IX binding to rDNA gene promoters (Figure 3(a)) was significantly decreased, and rRNA 45S transcript levels (Figure 3(b)) were underrepresented in hypoxic cells. As expected, the transcripts of the HIF1*α* targets, CA IX and LDHA, were upregulated in hypoxic cells (Figure 3(b)). Accordingly, some hypoxic cells showed increased representation of CA IX/XPO1 complexes, as demonstrated by the appearance of yellow nucleoli or nucleolar spots in the merged immunofluorescence experiment shown in Figure 3(c). Treatment of hypoxic cells with leptomycin B led to disappearance of these complexes (Figure 3(c)) and to increased representation of CA IX on rDNA 45S genes (Figure 3(a)). The levels of rRNA 45S transcripts were extremely low under this condition (Figure 3(b)), in agreement with the loss of UBF1 binding (Figure 3(a)).

Exposure of cells to stresses, including hypoxic condition, is characterized by altered cellular metabolism and attenuated protein synthesis [32]. In order to cope with this stressful condition and to support survival, glucose-deprived and hypoxic cells may decrease transcription of 45S rDNA genes by RNA polymerase I [33, 34], which is among the most energy-demanding processes in a cell, normally accounting for up to 60% of total cellular transcription [35]. While the increases in CA IX protein levels may represent a survival strategy for the hypoxic cells, concentration of a CA IX pool to complexes with exportins, and potentially with additional proteins of the nuclear trafficking, on one hand may represent a mechanism to decrease the energy-demanding 45S rDNA gene transcription. On the other hand, this can also represent a mechanism for CA IX storage in the nucleolar compartments, for putative adaptation of cells to the chronic hypoxia. XPO1 (also known as CRM1 homolog) function is commonly related to nuclear export of both proteins and RNAs [36, 37]; since XPO1 was also heavily redistributed to nucleoli in the hypoxic cells, this may represent a mechanism to decrease the nuclear export of mature ribosomal RNAs and of proteins exiting nuclear compartments during hypoxia, thus allowing the cells to react to nucleolar stress.

**Figure 4:**
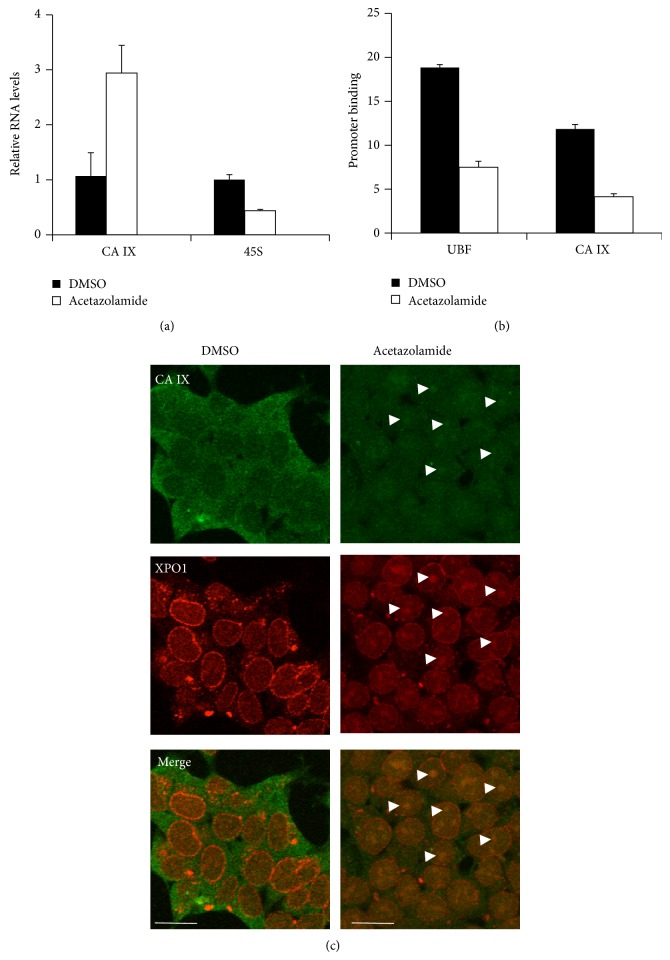
Cellular acidosis induced by acetazolamide mimics the hypoxia-induced relocalization of CA IX from 45S rDNA sites to nucleolar complexes with XPO1. (a) Analysis of CA IX mRNA and 45S rRNA transcripts in cells exposed to carrier DMSO and to acetazolamide. Transcript levels were evaluated via quantitative RT-PCR analysis from triplicate samples. Calculated *P* values for pairwise comparisons of qRT-PCR data were in the 0.003 to 0.03 range (Student's *t*-test). (b) Cells, treated as in (a), were subjected to chromatin immunoprecipitation analysis of CA IX and UBF1 binding to 45S rDNA precursor gene clusters in a triplicate set of samples. Calculated *P* values for pairwise comparisons of ChIP data were in the 0.03 to 0.01 range (Student's *t*-test). (c) Cells subjected to the same treatments, as in (a) and (b), were fixed, permeabilized, and exposed to antibodies for CA IX (green) and XPO1 (red) immunofluorescence analysis. Confocal images from representative fields were taken. White arrowheads show some representative enrichments of CA IX and XPO1 in nucleolar compartments. White bars: 10 *μ*m.

Thus, CA IX was able to interact with 45S rDNA gene promoters in normoxic cells, in which CA IX/XPO1 complexes were underrepresented or absent [17]. Acute hypoxia resulted in decreased binding of CA IX to the ribosomal gene clusters in nucleoli and in decreased expression of 45S rRNA precursor transcripts. Acetazolamide, a general inhibitor of CA enzymes, is known to induce metabolic acidosis in cultured cells and in mammals, including rats and humans [38–40]. Accordingly, acetazolamide induces normoxic induction of HIF1 alpha in cultured cells [38]. Thus, acetazolamide treatment should mimic what is actually observed during acute hypoxia, as shown in previous experiments. We then treated HEK293 cells with acetazolamide which, as expected, upregulated CA IX transcripts (Figure 4(a)). In a complementary manner, 45S rRNA transcript levels were downregulated. These results were associated with decreased binding of CA IX to 45S rDNA gene promoters (Figure 4(b)). Accordingly, as shown in Figure 4(c), treatment with acetazolamide induced, in HEK293 cells, the relocalization of CA IX and XPO1 in nucleoli. Thus, a drug-induced activation of cellular responses to hypoxic stress faithfully recapitulated the molecular events associated with regulated binding of CA IX to 45S rDNA promoters, adding support to the role of XPO1 in preventing this interaction during hypoxia. UBF1 binding to 45S rDNA genes was increased in hypoxic cells (Figure 3(a)), in accordance with a previous report [34], in which restriction of rRNA biosynthesis during hypoxia was associated with increased occupancy of UBF1 on 45S ribosomal gene clusters. Interestingly, UBF1 binding to 45S rDNA genes was decreased in acetazolamide-treated cells (Figure 4(b)), since cellular acidosis may not recapitulate in full the molecular effects, raised by acute hypoxia, on UBF1 interaction with rDNA genes.

## 4. Conclusions

The data reported in this paper for the first time describe a function for CA IX and for XPO1, one of its major interactors, in nucleoli. Firstly, we showed evidence for regulated binding of CA IX to nucleolar 45S rDNA genes in human cells. Additionally, we revealed, in hypoxic cells, a XPO1-based decoy mechanism, resulting in CA IX removal from nucleolar chromatin. The presence of the CA IX/XPO1 complexes, highly enriched in extrachromatinic nucleolar sites, was consistently associated with decreased transcription of 45S rDNA genes, both in hypoxic cells and in cells treated with acetazolamide, in which cellular acidosis was generated, as a consequence of general CA inhibition. We believe that these events are related to cellular responses to both hypoxic and hypoxia-induced nucleolar stresses, in which survival mechanisms should be activated. These mechanisms, in first instance, should attenuate energy-demanding processes, such as ribosome biogenesis and translation, to allow cell survival in the progression towards the adaptation to the stressful conditions. The contribution of CA IX and its complexes with XPO1 to such responses may reveal novel mechanisms in cell and cancer biology.

## Abbreviations

CA: Carbonic anhydrase
ChIP: Chromatin immunoprecipitation
EGFP: Enhanced green fluorescent protein
LMB: Leptomycin B
NES: Nuclear export signal
NLS: Nuclear localization sequence
qPCR: Quantitative PCR
qRT-PCR: Quantitative reverse-transcriptase PCR
rDNA: Ribosomal DNA precursor gene
rRNA: Ribosomal RNA.
